# An optimized deep learning architecture for breast cancer diagnosis based on improved marine predators algorithm

**DOI:** 10.1007/s00521-022-07445-5

**Published:** 2022-06-08

**Authors:** Essam H. Houssein, Marwa M. Emam, Abdelmgeid A. Ali

**Affiliations:** Faculty of Computers and Information, Minia University, Minia, Egypt

**Keywords:** Breast cancer classification, Deep learning, Transfer learning, Convolutional neural network, Marine predators algorithm, Opposition-based learning, Hyperparameters optimization

## Abstract

Breast cancer is the second leading cause of death in women; therefore, effective early detection of this cancer can reduce its mortality rate. Breast cancer detection and classification in the early phases of development may allow for optimal therapy. Convolutional neural networks (CNNs) have enhanced tumor detection and classification efficiency in medical imaging compared to traditional approaches. This paper proposes a novel classification model for breast cancer diagnosis based on a hybridized CNN and an improved optimization algorithm, along with transfer learning, to help radiologists detect abnormalities efficiently. The marine predators algorithm (MPA) is the optimization algorithm we used, and we improve it using the opposition-based learning strategy to cope with the implied weaknesses of the original MPA. The improved marine predators algorithm (IMPA) is used to find the best values for the hyperparameters of the CNN architecture. The proposed method uses a pretrained CNN model called ResNet50 (residual network). This model is hybridized with the IMPA algorithm, resulting in an architecture called IMPA-ResNet50. Our evaluation is performed on two mammographic datasets, the mammographic image analysis society (MIAS) and curated breast imaging subset of DDSM (CBIS-DDSM) datasets. The proposed model was compared with other state-of-the-art approaches. The obtained results showed that the proposed model outperforms the compared state-of-the-art approaches, which are beneficial to classification performance, achieving 98.32% accuracy, 98.56% sensitivity, and 98.68% specificity on the CBIS-DDSM dataset and 98.88% accuracy, 97.61% sensitivity, and 98.40% specificity on the MIAS dataset. To evaluate the performance of IMPA in finding the optimal values for the hyperparameters of ResNet50 architecture, it compared to four other optimization algorithms including gravitational search algorithm (GSA), Harris hawks optimization (HHO), whale optimization algorithm (WOA), and the original MPA algorithm. The counterparts algorithms are also hybrid with the ResNet50 architecture produce models named GSA-ResNet50, HHO-ResNet50, WOA-ResNet50, and MPA-ResNet50, respectively. The results indicated that the proposed IMPA-ResNet50 is achieved a better performance than other counterparts.

## Introduction

Breast cancer is a familiar frequent malignancy in females and the second most common leading cause of death in women. The global occurrence of breast cancer has increased over time, and more cases have been reported every year. In comparison to other malignancies, it is more common in women. If this disease is not detected early, it might lead to death [1]. Early diagnosis improves the chances of successful therapy and survival, but its diagnosis is time-consuming and frequently results in an agreement between pathologists. Computer-aided diagnosis (CAD) systems can improve diagnostic accuracy. Breast cancer can be classified as benign (non-hazardous) or malignant (threatening). Early detection of breast lesions and the distinction of malignant from benign lesions is critical for breast cancer prognosis [2]. Breast cancer affects approximately a million women globally each year, accounting for more than 25% of all female cancer occurrences. There is a tremendous need for early-stage development of new breast cancer methods with this exponential expansion. This motivates researchers to develop innovative methods for obtaining rapid and accurate diagnoses, ultimately extending patients’ lives [1]. Reviewing preliminary diagnostic information and gathering relevant data from previous information is key to detecting this disease early and reliably. Medical imaging and deep learning (DL) techniques will aid this procedure. Medical imaging performs a significant part in clinical illness diagnosis, therapy evaluation, and the detection of anomalies in many body components, including eye [3], lungs [3], brain [4], breast [5], and stomach [6]. Medical image research aims to classify an organ’s location, size, and characteristics in question, which is regarded as a viable way to obtain usable information from vast amounts of data. The most effective process to identify breast cancer is through medical imaging, including mammography images, histopathology images, magnetic resonance imaging (MRI), ultrasound, and thermography images [7].

Thermography images, also known as thermal imaging, have shown enormous promise in the early detection of breast cancer over the previous decade. Thermography images can help with other types of diagnosis by providing information about physiological changes [8].

Ultrasound imaging is another method for breast cancer detection that young women mostly use it. Noise levels might cause the process to fail when it attempts to detect microcalcifications and deeper breast tissue [9].

In addition to ultrasound and thermography screening, MRI is another approach for detecting cancer cells early. MRI uses magnetic instead of X-rays to produce extremely accurate three-dimensional (3D) transverse imaging [7].

Another imaging modality is called mammography images. Mammography is a very well and generally used approach for breast cancer screening, and it is the only image type that has been shown to decrease breast cancer mortality significantly [10]. It is an x-ray test image regarded as a reliable and accurate method for detecting breast cancer. [11]. In this paper, we used mammography images for breast cancer classification.

CAD systems have been used to aid clinicians in interpreting medical imaging to improve disease diagnosis. The critical role of a CAD system is feature extraction. Traditional feature extraction techniques have disadvantages because they lack flexibility [10]. Recently, DL approaches have been presented for breast cancer diagnosis.

DL is a category of machine learning and artificial intelligence that focuses on a complicated structure of image features due to its capacity to learn autonomously. DL approaches use various recently developed models to improve feature extraction from data. These models have been used in various medical fields [7, 12]. DL is composed of multilayer neural networks (NNs) that use raw input images to generate a hierarchical feature structure. Stack autoencoders, deep-Boltzmann machines, and convolution NNs (CNNs) are examples of common DL algorithms [13].

CNNs have had significant success in biomedical imaging, such as mitosis cell detection from microscopic images, tumor detection, segmentation of neural laminae, skin disease, immune cell detection and classification, mass detection, COVID-19 prediction [14] and classification in mammograms [15]. CNNs are a popular technique for object detection and image classification that involves layer-wise automatic feature extraction. The preparation of CNNs for classification objectives depends on the knowledge of hyperparameter tuning. Hyperparameters for each layer are different. Few studies, such as [16], have recognized the importance of the hyperparameters in achieving high performance with CNN architectures and the need to consider them as an optimization problem. Because a CNN model’s performance is determined by these hyperparameters [17], they need to be fine-tuned to achieve great results. The selection of hyperparameter values is frequently based on a mix of human expertise, trial and error, or a grid search method [18]. Training can take more days because of the computationally expensive core of CNN designs. Since the number of combinations grows exponentially with the increase in hyperparameters, the grid search method is usually not suited for CNN models. Tuning hyperparameters is a time-consuming effort for researchers; the number of layers of CNNs is increasing daily to cope with vast and complex datasets. It is not suitable to optimize hyperparameters manually at a reasonable cost. To improve them, different researchers have adopted different techniques. Some of them accepted their outcome, whereas others did not [17].

Thus, automatic hyperparameter optimization for CNN models is critical [19]. Meta-heuristic algorithms have significantly influenced hyperparameter optimization in several fields. They have been developed to address different real-world problems and have gained enormous interest in classification problems. Because meta-heuristic algorithms have great performance and are straightforward to implement, researchers have widely proved their experience to handle many types of challenging optimization issues in engineering, communications, industry, and social sciences [20]. In addition, they have been employed in biological information [21], chemical information [22], feature selection [23], task scheduling in cloud computing [24], image segmentation [25], global optimization [26], and as well as cost-effective emission dispatch [27]. There are various meta-heuristic algorithms, of which the marine predators algorithm (MPA) [28]. MPA is one of the most recent algorithms, released in 2020 by Faramarzi et al., [28], showing more quality results than several classical and the latest counterparts on various mathematical and engineering benchmark problems. Faramarzi et al. applied twenty-nine test functions to evaluate the MPA performance, and it showed high performance in different optimization problems. MPA has many advantages, including requiring the fewest amount of adjustable parameters, being simple to implement with powerful search capability, and flexible in altering the basic MPA version [29].

However, all meta-heuristic algorithms should strike a balance between exploration and exploitation; other solutions can be stuck in optimal solutions or fail to converge [30, 31]. Premature convergence is a problem for the MPA. As it splits the optimization iterations into three parts, the first part is only for exploration. The second part serves as a means of transitioning from exploration to exploitation. The third part is specified for the exploitation phase; this could affect the search process by causing the population to get stuck in local optimal. Accordingly, like other metaheuristic algorithms, the MPA has been improved. The no-free-lunch theorem states that no single method can solve all types of optimization problems. As a result of hybridization, various features can be combined into a single algorithm to address multiple challenges.

Thus, hybridization of many techniques from various scientific domains is required. Hybridization merges the benefits of various algorithms to create a more powerful, high-performing version with a promise of higher accuracy and performance. Notwithstanding MPA’s success in search processes, it might be improved in various areas to demonstrate its usefulness on more complex optimization projects. To enhance the MPA, we hybrid it with the opposition-based learning (OBL) strategy [32] to produce solutions from probable regions to explore the search space more thoroughly. OBL is one of the most efficient approaches to improve meta-heuristic algorithms [33]. It is merged with meta-heuristic algorithms in multiple ways to improve explorative searchability. After that, the proposed Improved Marine Predators Algorithm (IMPA) is used to optimize the CNN’s hyperparameters. As a result, automatic hyperparameter optimization for CNN architectures is critical. This paper proposes an improved meta-heuristic algorithm called IMPA using the OBL strategy to optimize the hyperparameters of the CNN architecture used for breast cancer classification. The proposed method is IMPA-ResNet50, which depends on a pretrained CNN model named ResNet50 to diagnose breast cancer from two mammographic datasets, the mammographic image analysis society (MIAS) and curated breast imaging subset of DDSM (CBIS-DDSM) datasets, using transfer learning (TL). The main contributions of this paper are as follows:

- This paper proposes a diagnostic model for breast cancer.
- The proposed model is IMPA-ResNet50, which is based on TL and uses a pretrained CNN model called ResNet50, along with an enhanced optimization algorithm called IMPA.
- OBL has been used to improve the performance of the MPA.
- IMPA, an improved version of the original MPA based on OBL, is proposed to optimize the hyperparameters of CNN architecture.

The remainder of this paper is structured as follows: Sect. 2 presents some literature reviews. Section 3 explains the MPA, the OBL strategy, CNNs, and TL. Section 4 presents the improved version of the MPA (the IMPA method). Section 5 introduces details of the proposed model. The experimental results and performance analysis including the limitation of the proposed model are explained in Sect. 6. Finally, Sect. 7 concludes this paper and presents future directions.

## Literature review

This section provides a summary of previous work on breast cancer diagnosis. In [34], the authors presented a review of some works on breast cancer classification, and from this review, they conclude that CNNs achieve higher accuracy than multilayer perceptron (MLP) NNs. The authors in [15] proposed a DL technique based on TL. They used three pretrained models, namely GoogLeNet, VGGNet, and ResNet, to classify malignant and benign cells. They evaluate their approach on cytology images to see how well it works. In addition, in [35], the authors presented a deep-CNN model that incorporated TL to avoid overfitting occurring when dealing with small datasets. They evaluated the presented model’s performance using four datasets: DDSM, INbreast, BCDR, and MIAS datasets. The DDSM dataset showed 97.35% accuracy and 0.98 area under the curve (AUC). The INbreast dataset yielded 95.5% accuracy and 0.97 AUC, whereas the BCDR database yielded 96.67% accuracy and 0.96 AUC.

The researchers in [36] presented an approach called BDR-CNN-GCN that combines a graph-convolutional network (GCN) with a CNN. A basic eight-layer CNN was used that was integrated with batch normalization and dropout layer. The final BDR-CNN-GCN model was developed by combining this model with a two-layer GCN. The performance of this model was tested on the MIAS dataset, achieving 96.10% accuracy. Furthermore, in [37], the researchers presented a CNN Inception-v3 model that was trained on 316 images, yielding 0.946 AUC, 0.88 sensitivity, and 0.87 specificity. In addition, in [38], the authors proposed a classification method using CNN and TL. The purpose of the paper was to assess how eight fine-tuned pretrained models performed. In [39], the authors proposed a hybrid classification model: Alexnet, Mobilenet, and Resnet50. They achieved 95.6% accuracy from these hybridized models. In [40], the researchers used four different CNN architectures, namely InceptionV3, VGG16, ResNet50, and VGG19, including 5000 images, for model training (benign: 2500 and malignant: 2500). In addition, the prediction models were tested by 1007 images (benign: 788 and malignant: 219).

In the same context, in [41], the researchers proposed a TL model for classifying histopathological breast cancer images. The authors used the ResNet-18 model as a backbone model with a block-wise fine-tuning method. The performance of the model was improved using data augmentation techniques and global contrast normalization. In addition, in [42], the authors proposed a classification method for thermal images, which combines thermal images of different views using a CNN model. The method achieved 97% accuracy and 0.99 AUC, with a 100% specificity and 83% sensitivity. In [2], the authors presented a DL framework (DenseNet) that extracts image features and feeds them into a fully connected (FC) layer to classify cancerous and benign cells. The efficiency of the technique was evaluated through hyperparameter adjustment. In [43], the authors proposed a deep classification algorithm for mammography imaging named CNNI-BCC (CNN improvement for breast cancer). The CNNI-BCC model classifies breast images into malignant, benign, and healthy. They achieved 90.50% accuracy and 90.71% specificity. In [44], the authors demonstrate that TL provides improved performance. They employed Inception-v4 [45] pretrained with the ImageNet and DDSM datasets.

Furthermore, in [46], the authors used AlexNet, along with the support vector machine (SVM), to improve classification accuracy and using data augmentation techniques to increase input images. Performance is evaluated on two datasets: DDSM and CBIS-DDSM. The accuracy of the method is 71.01%, and when using SVM, the accuracy becomes 87.2%. In addition, in another work [47], a deep-CNN was proposed in which an MLP was employed in the FC layer to classify mammography images into benign, malignant, and normal. The authors employed a bilateral filter with vector grid computing to maintain edge information in the preprocessing step. They also use hyperparameter tuning to evaluate performance. The results demonstrate that hyperparameter tuning of the final layers produces 96.23% overall accuracy and 97.46% average accuracy. In addition, in [48] used the CBIS-DDSM dataset to develop an automatic mammogram classification method based on TL and data augmentation. ResNet was fine-tuned to produce good results, and achieved accuracy was 93.15%.

To be specific, despite the encouraging results achieved by the CNN architectures in detecting breast cancer, the large number of hyperparameters is a barrier to attaining improved results. Thus, the use of hyperparameter optimization for CNN architecture is essential to enhance the performance of CNNs. In this paper, an optimized CNN model based on an improved MPA (IMPA) algorithm was proposed for breast cancer classification, which may aid health professionals in breast cancer diagnosis.

## Preliminaries

This section explained the concept, the mathematical representation of the marine predators algorithm (MPA), along with the opposition-based learning (OBL) strategy, the convolutional neural networks (CNNs) architecture, and transfer learning (TL).

### Marine predators algorithm

The motivation of MPA appears from the general foraging operation in ocean predators as well as predator-prey communications. In this scenario, a predator optimizes encounter rates to increase the chances of surviving in natural surroundings. MPA uses Lévy flight and Brownian motion to do a search using two simple random walk methods. The Lévy flight is usually performed in meta-heuristic algorithms and is most effective to avoid solution stagnation by executing a constructive search in local area [49]. Also, Brownian motion is a well-known global search instrument. The designers of MPA merged the search effectiveness of Lévy and Brownian motion to increase the trade-off scale through exploration and exploitation [50].

MPA initializes the search by randomly determining $N_n$ search agents using Eq. (1):

1 $$\begin{aligned} x^0_i=lb_i+ \dot{q} \times (ub_i-lb_i), i\in \{1,2,\ldots ,N_n\} \end{aligned}$$

where $\dot{q}$ is a random number in [0,1], $lb_i$ and $ub_i$ are lower and upper bounds. During initialization and the basic population matrix, another $N_n\times \mathrm{Dim}$ matrix is generated, including search agents with best fitness values, $N_n$ and Dim indicate population size and dimensions. MPA refers to it as Elite:

2 $$\begin{aligned} \mathrm{Elite}=\begin{bmatrix} X_{1,1}^{I} &{} X_{1,2}^{I} &{} \ldots &{} X_{1,\mathrm{Dim}}^{I}\\ X_{2,1}^{I} &{} X_{2,2}^{I} &{} \ldots &{} X_{2,\mathrm{Dim}}^{I}\\ \vdots &{}\vdots &{}\vdots &{}\vdots \\ X_{N_n,1}^{I} &{} X_{N_n,2}^{I} &{} \ldots &{} X_{N_n,\mathrm{Dim}}^{I} \end{bmatrix}_{N_n \times \mathrm{Dim}} \end{aligned}$$

where $X^{I}$ denotes a vector with the highest fitness.

Prey is similar to Elite. Predators utilize it to update their positions. The initialization generates the initial prey, from which the fittest one creates the Elite in a single term. The Prey is depicted in the following way:

3 $$\begin{aligned} \mathrm{Prey}=\begin{bmatrix} X_{1,1} &{} X_{1,2} &{} \ldots &{} X_{1,\mathrm{Dim}}\\ X_{2,1} &{} X_{2,2} &{} \ldots &{} X_{2,\mathrm{Dim}}\\ \vdots &{}\vdots &{}\vdots &{}\vdots \\ X_{N_n,1} &{} X_{N_n,2} &{} \ldots &{} X_{N_n,\mathrm{Dim}} \end{bmatrix}_{N_n \times \mathrm{Dim}} \end{aligned}$$

$X_{i,j}$ indicates the $j_{\mathrm{th}}$ dimension of the $i_{\mathrm{th}}$ prey. These two matrices are crucial in the optimization process.

Following initialization, the primary iterative search method begins, which is divided into 3 stages that simulate various situations among predator and the prey while devising different search techniques. The three stages are based on iterations $it \in \{1,2,3\ldots it_{\mathrm{max}}\}$ where $it_{\mathrm{max}}$ denotes the maximum iterations. MPA refreshes potential solutions during these stages.

*Stage 1: High velocity* ($it<\frac{it_{\mathrm{max}}}{3}$) This simulates the situation in which the prey is outrunning the predator. This approach reinforces exploration and consumes more of the prior iterations. The mathematical representation of this rule is carried out by Eq. (4):

4 $$\begin{aligned} \begin{aligned} \overrightarrow{\text {stepsize}}_{j}&=\mathbf {R}_{r} \times \left( \overrightarrow{\text {Elite}}_{j}-\mathbf {R}_{r} \times \overrightarrow{\text {prey}_{j}}\right) , j \in \{1,2,\ldots ,N_n\} \\ \overrightarrow{\text { Prey }}_{j}&=\overrightarrow{\text { Prey }}_{j}+P \cdot \mathbf {R} \times \overrightarrow{\text {stepsize}}_{j}\\ \end{aligned} \end{aligned}$$

where $R_r$ is a random number between [0,1] relies on normal distribution identify the Brownian motion. P is a constant equal to 0.5, and *R* is a vector of uniform random numbers in [0, 1]. The speed of predators and prey is great during this stage, which aids in the exploration of far-flung areas of the search space.

*Stage 2: Unit velocity* ($\frac{1}{3}it_{\mathrm{max}}<it<\frac{2}{3}it_{\mathrm{max}}$) Prey and predator move at equal speed. The transmission step is transitioned from exploration to exploitation in this phase. As a result, the population is separated into prey as exploration utilizing Lévy motion and predator exploiting Brownian motion. The first half of population identified by Eq. (5) and the second half using Eq. (6).

5 $$\begin{aligned} \begin{aligned} \overrightarrow{\mathrm{stepsize}_{j}}&=\mathbf {R}_{\mathrm{levy}} \times \left( \overrightarrow{\text {Elite}}_{j}-\mathbf {R}_{levy} \times \overrightarrow{\mathrm{prey}_{j}}\right) , j \in \{1,2,\ldots ,N_n/2\} \\ \overrightarrow{\mathrm{prey}}_{j}&=\overrightarrow{\mathrm{prey}}_{j}+P \cdot \mathbf {R} \times \overrightarrow{\mathrm{stepsize}_{j}} \end{aligned} \end{aligned}$$

where $\mathbf {R}_{\mathrm{levy}}$ is a random number relies on the Lévy. The multiplication of $\mathbf {R}_{\mathrm{levy}}$, $\mathrm{prey}_{j}$ imitates the predator motion in Lévy, whereas adding the step size to prey position affects prey movement.

6 $$\begin{aligned} \begin{aligned} \overrightarrow{\mathrm{stepsize}_{j}}&=\mathbf {R}_{r} \times \left( \mathbf {R}_{r} \times \mathbf {\mathrm{Elite}}_{j}-\mathbf {\mathrm{Prey}}_{j}\right) , j \in \{N_n/2,\ldots ,N_n\} \\ \overrightarrow{\mathrm{prey}}_{j}&=\overrightarrow{\text {Elite}}_{j}+P *{\hat{\mathrm{CF}}} \times \overrightarrow{\mathrm{stepsize}_{j}} \\ \end{aligned} \end{aligned}$$

where $\hat{\mathrm{CF}}$ is a parameter that control step size and is calculated using Eq. (7):

7 $$\begin{aligned} {\hat{\mathrm{CF}}}=\left( 1-\frac{\mathrm{it}}{\mathrm{it}_{\mathrm{max}}}\right) ^{\left( 2\frac{t}{\mathrm{it}_{\mathrm{max}}}\right) } \end{aligned}$$

*Stage 3: Low velocity* ($\mathrm{it}>\frac{2}{3}\mathrm{it}_{\mathrm{max}}$): The population can be modified by the Lévy flight using Eq. (8):

8 $$\begin{aligned} \begin{aligned} \overrightarrow{\mathrm{stepsize}_{j}}&=\mathbf {R}_{\mathrm{levy}} \times \left( \mathbf {R}_{\mathrm{levy}} \times \overrightarrow{\mathrm{Elite}}_{j}-\mathbf {\mathrm{Prey}}_{j}\right) , j \in \{1,\ldots ,N_n\} \\ \overrightarrow{\mathrm{prey}}_{j}&=\overrightarrow{\text {Elite}}_{j}+P *{\hat{\mathrm{CF}}} \times \overrightarrow{\mathrm{stepsize}_{j}}\\ \end{aligned} \end{aligned}$$

MPA also employs a theory in marine predators known as eddy formation or Fish Aggregating Devices (FADs), where the predators contemplate longer jumps in different positions in quest of more food to infuse variation into possible solutions using Eq. (9):

9 $$\overrightarrow{prey}_{j}= \left\{ {\begin{array}{*{20}l} \overrightarrow{prey}_{j}+CF \big [ lb_i+r \times (ub_i-lb_i) \big ] \times \ell&r\le FADs \hfill \\ \overrightarrow{prey}_{j}+[FADs(1-r)+r](\overrightarrow{prey}_{r1}-\overrightarrow{prey}_{r2})&\text {else} \hfill \\ \end{array} } \right.$$

where $\overrightarrow{\mathrm{prey}}_{j}$, $\overrightarrow{\mathrm{prey}}_{r1}$, and $\overrightarrow{\mathrm{prey}}_{r2}$ denotes vectors for *j*th candidate solution, a random finding solution, and another random finding solution, respectively; where, *r* indicates a random number in [0,1], FADs is a constant equal to 0.2, $\ell$ a binary vector includes zero and one.

### Opposition-based learning

OBL [32] is a helpful approach in avoiding staleness in competitor solutions [51]. It is an essential concept, which enhances search mechanism exploitation. In meta-heuristic algorithms, convergence occurs almost rapidly when the primary solutions are near the ideal position; otherwise, late convergence is expected. By exploring opposite search zones close to the global optimal, the OBL strategy produces better results. The OBL strategy operates by searching the search space in two directions. These two directions are defined by one solution, whereas the other direction is defined by the opposite solution. The OBL strategy then chooses the best direction from all solutions [52].

- *Opposition number* The concept of opposite numbers may be explained by describing OBL. It is defined by considering $Y_0$ as a real number $\in [u, p]$. The opposite number of $Y_0$ is denoted using Eq. (10) [52]:
  10 $$\begin{aligned} \overline{\mathrm{Y}}_{0}={u + p} - {Y_{0}}. \end{aligned}$$
  The opposite number in dimensional (M) space is calculated using Eq. 11 and Eq. 12:
  11 $$\begin{aligned} Y&={Y_{1},Y_{2},Y_{3},\ldots ,Y_{M}} \end{aligned}$$
  12 $$\begin{aligned} \overline{\mathrm{Y}}&={[\overline{\mathrm{Y}}_{1}, \overline{\mathrm{Y}}_{2}, \overline{\mathrm{Y}}_{3}, . . .,\overline{\mathrm{Y}}_{M}]} \end{aligned}$$
  The items in $\overline{\mathrm{Y}}$ are represented by Eq. (13):
  13 $$\begin{aligned} \overline{\mathrm{Y}}_{k}={u_{q}+p_{q}} - {Y_{q}} \text { where } q = {1, 2, 3, \ldots , M} \end{aligned}$$
- *Opposition-based optimizations* The opposite item $\overline{\mathrm{Y}}_{0}$ is changed with the corresponding solution $Y_0$ throughout the fitness function. If $f_t(Y_0)$ is better than $f_t(\overline{\mathrm{Y}}_{0})$, $Y_0$ is constant; oppositely, $Y_0$ = $\overline{\mathrm{Y}}_{0}$. As a result, the solutions have been modified in terms of the best value of Y and $\overline{\mathrm{Y}}$ [53].

### Convolutional neural networks

**Fig. 1:**
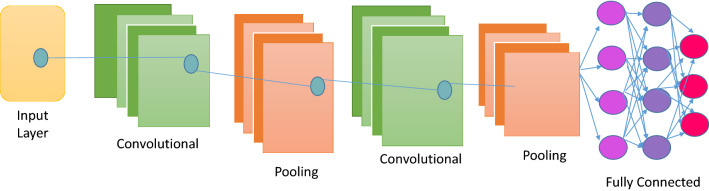
CNN standard architecture [7]

The main structure of every CNN architecture will be discussed in this subsection. They are a type of deep NNs that is used to recognize and classify images. Recently, CNNs have become an essential method in image analysis, especially when recognizing or detecting faces, text, and medical imaging [13]. CNNs have been successfully used for image classification and segmentation since their initial development in 1989 [54], and they are designed to work as the human brain does in visual perception: they include layers of “neurons” that only react to neurons in their immediate environment. CNNs can extract the topological aspects of an image by concatenating three types of layers[55]: ***convolutional layers***, ***pooling layers***, and ***and FC layers.*** Figure 1, shows an example of CNN architecture [7].

- *Convolutional layers* These layers are structured into feature maps according to the local connection concept and weight distribution concept. A weight group known as a filter bank connects all neurons in a feature map to local patches at the previous stage. All units share a filter row on a feature map. For various feature maps, different filter banks are used. The reason for local connection and weight distribution is to reduce the number of parameters by exploiting the highly linked local pixel neighborhood, and local image characteristics are location-independent. Then, the summation of weights is passed on to an **activation function** such as Sigmoid [56] and rectified linear unit (ReLU) [57]. The activation function facilitates the nonlinear transformation of transmitted data to the following processing phases [58].
- *Pooling layer* As shown in Fig. 1, the pooling layer comes after the convolution layer. It employs a subsampling technique to integrate convolutional layer features comparable to a single layer (semantically). The primary goal of this layer is to reduce an image’s dimension (by close grouping pixels in a specific portion of the image into a single value) while highlighting its features. Some of the common popular kinds of operation performed on this layer are max pooling and main pooling [59].
- *Fully connected layer* The final layer of the CNN is a classifier, which decide the class of the input data based on CNN’s features discovered and extracted. The number of units in the FC layer is equal to the number of classes or classifications. [59].

**Fig. 2:**
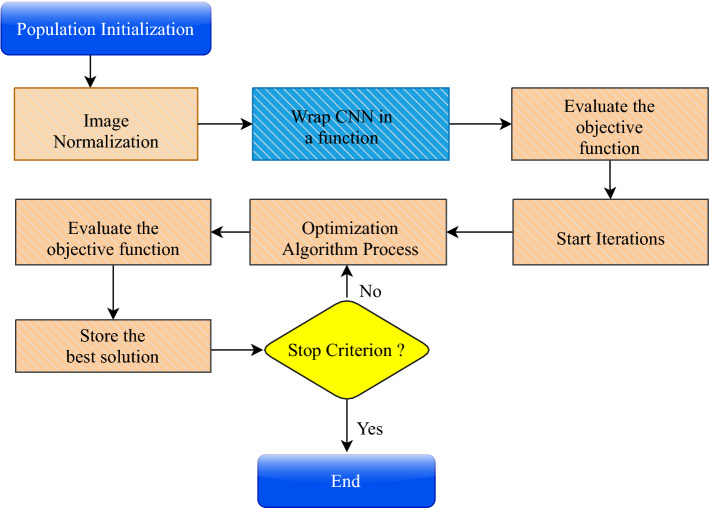
Block diagram of the standard process for hyperparameter optimization in a CNN using meta-heuristic algorithms

A CNN model depends on its hyperparameters, so to improve its accuracy, some researchers have proposed that these hyperparameters must be fine-tuned to achieve great results. Table 1 shows hyperparameters and their description related to CNN architecture. As mentioned before in Sect. 1, the meta-heuristic algorithms are widely regarded as excellent techniques to optimize the hyperparameters of the CNN architecture to increase its performance. Figure 2 presents the process of using an optimization algorithm to optimize a CNN’s hyperparameters.

**Table 1:** Hyperparameter description of CNN

Hyperparameter-name	Description
Learning rate	The initial learning rate for the CNN architecture is one of the significant hyperparameters that affect output performance. When the learning rate is low, the model requires more iterations
Number of hidden layer units	Expanding the number of hidden layer units enhances the model and reduces computational efficiency
Batch size	It refers to the number of sub-samples sent to the network for parameter updates
Dropout rate	A dropout is a regularization approach that reduces overfitting by enhancing validation accuracy and consequently generalizing power
Activation Function	Activation functions allow DL techniques to learn nonlinear prediction limits
Number of epochs	It is the number of times the entire training data is taken through the training process

As shown in Fig. 2, optimizing hyperparameters of a CNN starts by initializing the meta-heuristic algorithm’s population, and the number of hyperparameters determines the number of dimensions to optimize. Following that, images must be normalized before being fed to the CNN. The suggested technique puts the CNN architecture in a function so that it may be called later when the fitness function is assessed; at this point, hyperparameters will be optimized. Once the fitness function has been established, the iterations of the meta-heuristic algorithm’s positions are updated according to the algorithm employed. New solutions are assessed, and the best one is selected. Finally, the stop criterion is assessed; if not, the operation is continued to obtain new solutions.

### Transfer learning

TL is a key to improving the performance of a DL model on a small dataset, such as medical images. DL models need a lot of data, computing power, and time to train from scratch. To tackle these issues, pretrained models and fine-tuning (FT) are used. TL enables a DL architecture to learn efficiently from a dataset with fewer samples by transferring learned features from other DL architectures that have earlier learned from large datasets [60]. Using a pretrained network is one of the most well-known and frequently used strategies to deal with small datasets, such as ImageNet [61], used in the assigned tasks [62]. Several models have been pretrained on the ImageNet, such as AlexNet [63], VGG [64], ResNet [65], Inception [66], and DenseNet [67]. Feature extraction and Fine tuning (FT) are different approaches that TL can use. The feature extraction approach removes FL layers from an ImageNet-trained network while maintaining the remaining network layers, which comprise a sequence of convolution and pooling layers and are known as the convolutional part, a fixed feature extractor. Then, on top of the fixed feature extractor, any classifier can be added.

FT is to replace the FC layers of a pretrained model with new ones and retrain them on the input dataset and fine-tune the kernels in the pretrained model’s convolutional part using backpropagation. It is the same as the feature extraction method, except that the final layers of the frozen convolutional part are unfrozen. These layers are then retrained, along with the new classifier obtained via the feature extraction procedure. FT seeks to make the most abstract elements of the pretrained model more relevant to the new goal [19]. The steps for implementing these methods are as follows:

- The classifier of the pretrained model is removed.
- The pretrained model’s convolutional base is frozen.
- Add a new classifier and train it on the top of the convolutional part. Then, some layers of the convolutional part are unfreezing.
- Finally, the new classifier and the unfrozen layers are jointly trained.

## Improved marine predators algorithm

In this section, the proposed IMPA is explained. When examining the performance of the original MPA, it is clear that it does not adequately explore all search space solutions. Furthermore, because it divides the optimization phases into three discrete portions, it suffers from a convergence rate. Thus, the original version of the MPA is improved using the OBL strategy (IMPA) and used to optimize the hyperparameters of the pretrained CNN architecture. Algorithm 1 presents the pseudo-code for the IMPA. The MPA’s diversity was improved in the search process using the OBL strategy during the initialization phase to improve the search operation as follows:

14 $$\begin{aligned} \mathrm{Opp}_s = lb_a+ub_a-y_b, b\in {1,2,\ldots ,N_n} \end{aligned}$$

where $\mathrm{Opp}_s$ is a vector produced by applying OBL, $lb_a$, and $ub_a$ are lower and upper bounds of the *a*th component of Y, respectively.

The phases of the proposed IMPA are described in the next subsections:

### Initialization steps in IMPA

The IMPA starts by initializing its parameters: maximum iterations $t_{\mathrm{max}}$, population size $N_n$, FADs, *P*, and dimensions *Dim*. The MPA begins by initializing the first search agent $y^0$ and saving outputs. Then, the OBL strategy is applied to determine the $\mathrm{Opp}_{s}$ of the initial population by Eq. (14).

### Optimization processes

The optimization operation is divided into three phases as presented in Sect. 3.1. After completing these phases, the OBL strategy is used to calculate the fitness function for each solution in *y* and $\overline{y}$, the proposed method updates the global best solution by calculating and comparing the fitness of $y_b$ and $\mathrm{Opp}_s$.

### Final steps in IMPA

After finishing the optimization process, saving the memory, and updating Elite, and the FADs are calculated by Eq.(9). The proposed method selects the best solution.

### IMPA computational complexity

The IMPA’s time and space expenses are explained in this subsection as follows:

1. *Time complexity * The IMPA generates $N_n$ search agents with size *Dim*, and the initialization time complexity was $O(N_n \times \mathrm{Dim})$. In addition, the IMPA computes the fitness of each search agent as $O(It_{\mathrm{max}} \times N_n \times \mathrm{Dim})$, where $It_{\mathrm{max}}$ determines the maximum iterations. Furthermore, the IMPA requires $O(T_t)$ to perform $T_t$ number of its primary processes. So, the time complexity of the IMPA is represented by $O(It_{\mathrm{max}} \times T_t \times N_n \times \mathrm{Dim})$.
2. *Space complexity * The IMPA space complexity is $O(N_n \times \mathrm{Dim})$.

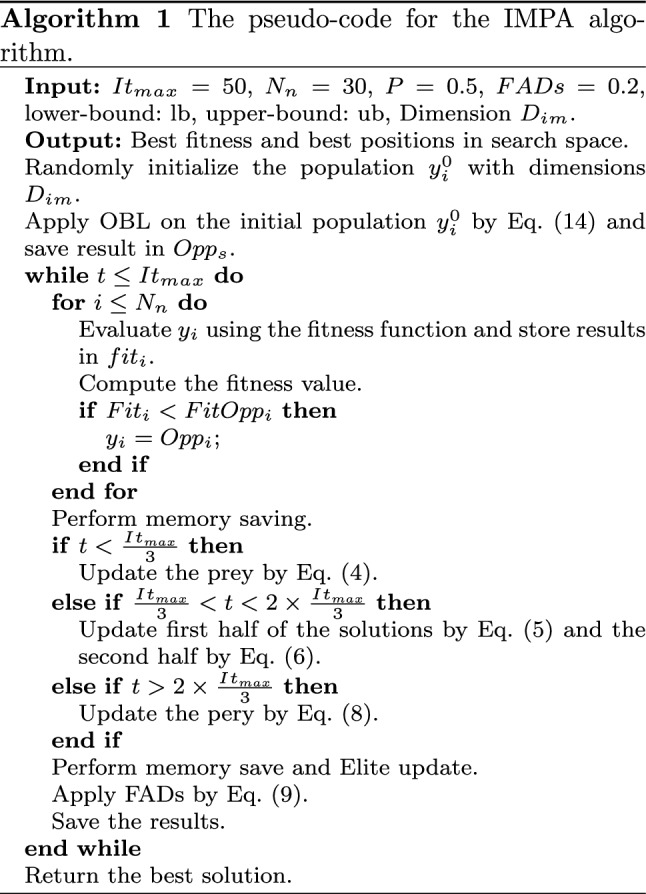

## The proposed IMPA-ResNet50 classification model

**Fig. 3:**
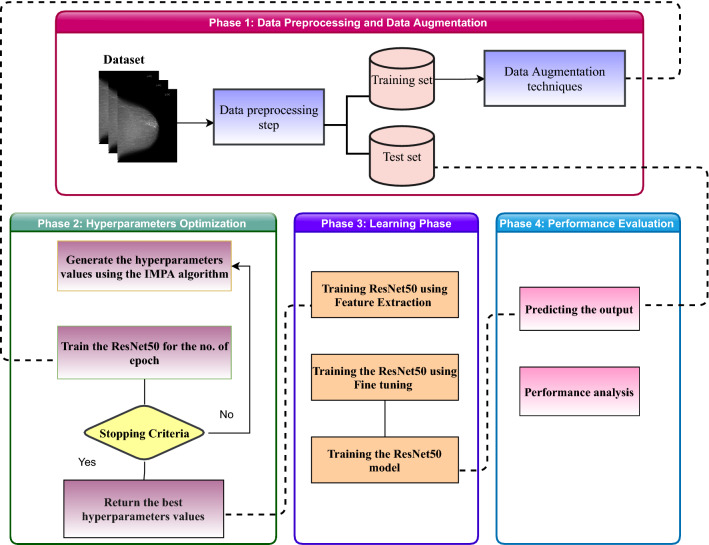
The proposed IMPA-ResNet50 architecture block-diagram phases

This section introduces the proposed IMPA-ResNet50 model based on TL from a pretrained CNN architecture. The pretrained model employed in this proposed model is ResNet50. The IMPA algorithm is used to optimize the hyperparameters of the pretrained CNN model to get the best performance of this model. The ResNet50 model was trained using TL methods after determining the best values for the parameters. After the model has been trained, it is verified using a different test set. The test set is then used to validate the fully trained model. The proposed model has split into four main phases, as shown in Fig. 3. These phases operate in this order:

1. *Phase 1* Data preprocessing and data augmentation.
2. *Phase 2* Hyperparameters optimization.
3. *Phase 3* The learning phase.
4. *Phase 4* The performance evaluation.

In the first phase, the datasets were enhanced and divided into two training and test sets. Also, multiple data augmentation procedures have been applied to increase training sets. The proposed model has been applied to two datasets, namely CBIS-DDSM and MIAS. In the second phase, the IMPA is used to optimize the hyperparameters in the pretrained CNN architecture (ResNet50). In the third phase, ResNet50 was completely trained using the values of the hyperparameters determined in the second phase that helped the architecture accurately diagnose the test set in the next phase. The phases of IMPA-ResNet50 will be presented in detail in the following sections.

### Phase 1: data prepossessing and data augmentation

In this phase, data preprocessing and data augmentation were applied to the two mammographic datasets. First, the images were enhanced by removing noise and resizing them to $224 \times 224$ resolution before applying data augmentation, thereby minimizing the storage capacity and reducing computational time. Second, multiple data augmentation procedures [68] have been applied to increase training sets, decrease overfitting, speed up the convergence process, and improve generalization. Here, data augmentation was implemented using the Keras ImageDataGenerator to enlarge the images of the dataset’s training set. Table 2 lists the used data augmentation approaches and their ranges.

**Table 2:** The data augmentation approaches and their ranges

Data-augmentation technique	Range
Shearing	0.1
Zooming	0.1
Width shift	0.3
Height shift	0.3
Rotation	15
Featurewise center	True
Featurewise standard deviation normalization	True
Fill mode	Reflect
Vertical flip	True
Horizontal flip	True

### Phase 2: hyperparameters optimization

The TL approach adopts the exact structure of the pretrained architecture after making small modifications. The most significant difference is that the classifier is replaced with a new one, requiring adjusting or adding several hyperparameter values. Tuning parameters of a CNN has a significant contribution to classification efficiency. As previously mentioned in Sect. 3.3, in this subsection, we determine hyperparameters that the proposed IMPA can optimize. In the proposed IMPA-ResNet50 model, eight hyperparameters are optimized: the learning rate, the batch size, the three dropout rates of the three dropout layers, and the number of units of the first three dense layers. As a result, the search space is eight-dimensional, with each point representing a combination of the eight hyperparameters.

### Phase 3: learning phase

Feature extraction and FT are performed to adapt the ResNet50 model to learn from the used datasets (CBIS-DDSM and MIAS). The convolutional base is unmodified in the feature extraction process, but the basic classifier is replaced by the newest one, which fits the datasets. The new classifier has eight layers: a flatten layer, four dense layers, and three dropout layers separating the dense layers. The IMPA is used to calculate the learning rate for the convolutional layer, the number of neurons in the first three dense layers that use the activation function (ReLU), and the rates of all dropout layers. The last dense layer has one neuron with a softmax function. After training the new classifier for some epochs, fine-tuning is performed by retraining the last two blocks of the convolutional part of ResNet50 integrating the new classifier.

### Phase 4: performance evaluation

Accuracy, sensitivity, specificity, precision, F-score, and AUC are the metrics used in this paper to assess the quality of the proposed method. The following is a summary of these metrics.

*Accuracy (Acc)* This determines how many cases have been correctly categorized. It is expressed by Eq. 15 [7]:

15 $$\begin{aligned} \begin{aligned}&\mathrm{ACC}=\frac{(\mathrm{TP} + \mathrm{TN})}{(\mathrm{TP} + \mathrm{TN} + \mathrm{FP} + \mathrm{FN})} \end{aligned} \end{aligned}$$

where TP defines true positive, TN defines true negative, FP defines false positive, and FN defines false negative.

*Sensitivity* ($S_{n}$) This analysis just displays how many of the total positive cases are only approximated correctly. This can be measured using Eq. 16 [7]:

16 $$\begin{aligned} \begin{aligned}&S_n=\frac{\mathrm{TP}}{(\mathrm{TP} + \mathrm{FN})} \end{aligned} \end{aligned}$$

*Specificity metric * ($S_p$) This metric shows how correct the overall pessimistic predictions are and how accurate the normal prediction is. It is expressed using Eq. 17 [7]:

17 $$\begin{aligned} \begin{aligned}&S_{p}=\frac{\mathrm{TN}}{(\mathrm{TN} + \mathrm{FP})} \end{aligned} \end{aligned}$$

*Precision metric* ($P_r$) This metric indicates how accurate the abnormal breast cancer prediction is. It is denoted using Eq. 18 [7]:

18 $$\begin{aligned} \begin{aligned}&P_{r}=\frac{\mathrm{TP}}{(\mathrm{TP} + \mathrm{FP})} \end{aligned} \end{aligned}$$

*Average F-score* F-score is a metric of the test accuracy, as expressed in Eq. (19) [69]:

19 $$\begin{aligned} \begin{aligned} F1_j&=\left\{ \frac{\mathrm{TP}_j}{\mathrm{TP}_j+\mathrm{FP}_j}\right\} \\ F1&= \frac{1}{q}\sum _{j=1}^{q} F1_j\\ \end{aligned} \end{aligned}$$

*AUC* The AUC indicates how well a model will perform in various scenarios, expressed using Eq. 20 [7]:

20 $$\begin{aligned} \begin{aligned}&\mathrm{AUC}=\frac{\sum R_{i}\left( I_{t} \right) - I_{t} ( I_{t} + 1)/2}{I_{t} + I_{f}} \end{aligned} \end{aligned}$$

where $I_t$ and $I_f$ represent the number of positive and negative images, and $R_i$ denotes the rate of the $i_{\mathrm{th}}$ positive image.

## Experimental results and performance analysis

This section describes and analyzes the results to validate the proposed IMPA-ResNet50 model’s performance for classifying mammography of breast cancer. This section is structured as follows: Sect. 6.1 presents the datasets used in this paper. Section 6.2 presents the platform used in this paper. Section 6.3 presents the experimental parameter settings for the IMPA. Section 6.4 presents the results of the CBIS-DDSM dataset, and Sect. 6.5 describes the results of the MIAS dataset. Section 6.6 presents the comparative results of the proposed IMPA-Resnet50 with four other meta-heuristic algorithms paring with the ResNet50 architecture: GSA-ResNet50, HHO-ResNet50, WOA-ResNet50, and MPA-ResNet50. Eventually, the limitations of the proposed model are illustrated in Sect. 6.7

### Dataset description

**Fig. 4:**
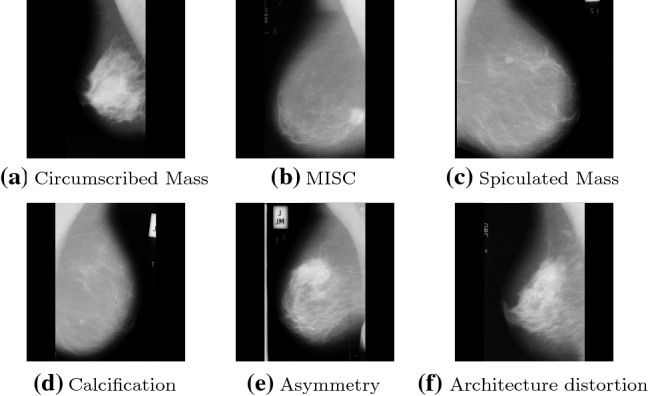
MIAS mammography Categories [70]

In this paper, two datasets were used to evaluate the proposed model including MIAS [70] and CBIS-DDSM [71] described as follows:

1. *CBIS-DDSM dataset*The CBIS-DDSM dataset is an upgraded and standard version of the DDSM mammogram dataset. Its images were decompressed and changed to the DICOM form for download. To process this dataset, we applied the guidance of CBIS-DDSM and transformed the DICOM format into PNG files to train the method to categorize images as benign or Cancer. The total number of images are 5283. Table 3 presents the specification of the dataset and its number of samples for the training and test sets.
2. *MIAS dataset* The MIAS dataset consists of 322 mammography images with a size $1024 \times 1024$. This dataset has two (abnormal: 113; normal: 209) classes. The abnormal category was divided into two classes: benign contains 65 images, and malignant contains 48 images. Each class in the MIAS dataset contains useful information. The abnormality type, such as calcifications, masses, and asymmetries, is identified in the data. This dataset is categorized into six categories shown in Fig. 1. After applying data augmentation techniques as mentioned in Sect. 5.1 on the MIAS dataset, the number of images increases, as reported in Table 4 (Fig. 4).

**Table 3:** Specifications of CBIS-DDSM dataset

Dataset	Class	No. of training samples	No. of test samples
CBIS-DDSM	Benign	1824	783
	Cancer	1873	803

**Table 4:** Specifications of MIAS dataset

Dataset	No. of training samples	No. of test samples	Total images
*MIAS*	904	386	1290
Normal-category			
830			
Abnormal-category			
460			

### Experimental platform

The IMPA and the IMPA-ResNet50 model are coded on Google Colaboratory [72] platform and implemented by Python 3 with Keras [73]. Keras is a high-level NN API that may be used with TensorFlow, CNTK, or Theano. It is the most popular DL framework. It was created for ease of use, and it has been able to conduct multiple tests and obtain findings as rapidly as possible, with the least amount of delay, allowing for adequate paper.

### Parameter settings

The parameters employed in the proposed model are listed in Table 5. The maximum number of iterations is 50, and the population size is 30, which are almost relative to our number of dimensions. The IMPA algorithm aims to optimize the initial learning rate parameter to fit in the optimal area. The range of the learning rate is between $1e^{-7}$ and $1e^{-3}$; it should be a small value in the fine-tuning method since the number of changes that will occur in the model must be quite small so that the features learned from the feature extraction method are not lost. The batch size value is between [1, 64]; the searching range of batch size is bounded by a lower bound of 1 and the upper bound of 64, and the dropout rates are in the range of [0.1, 0.9]; the searching range of dropout rates is bounded by a lower bound of 0.1 and the upper bound of 0.9 as a good value for dropout in a hidden layer is between 0.1 and 0.9, and the number of neurons is in the range of [50, 500]. The epochs training number of ResNet50 was chosen by experimenting with more than one value. When employing a number of epochs more than 30, the experiment determined that each IMPA’s training process takes exponential time. Also, when using less than 30 epochs, the results of the ResNet50 were not adequately accurate. As a result, the ResNet50 was trained using 30 epochs. The Dimension parameter of the IMPA denotes the number of hyperparameters that the proposed IMPA can optimize. It is set to 8 as they are 8 hyperparameters: the learning rate, the batch size, the three dropout rates of the three dropout layers, and the number of units of the first three dense layers.

**Table 5:** Parameter settings for IMPA

Parameter	Value
Maximum iteration numbers	50
Population size	30
Dimension	8
Learning rate	[1e−7, 1e−3]
Batch size	[1,64]
Dropout rate	[0.1,0.9]
Number of neurons	[50,500]
Maximum number of ResNet50 training epochs	30

### Analysis of IMPA-ResNet50 model for CBIS-DDSM dataset

This subsection presents the results of the proposed IMPA-ResNet50 model using hyperparameters determined by the IMPA according to the CBIS-DDSM dataset. In addition, it presents the comparison among other studies and related work. Furthermore, to demonstrate the effectiveness of the IMPA in determining the best values for the hyperparameters of the ResNet50 model that can achieve the most significant accuracy, we compare it with the ResNet50 model based on setting the hyperparameters of the ResNet50 manually, i.e., randomly chosen.

Table 6 presents the results of the IMPA-ResNet50 model on the CBIS-DDSM dataset evaluated in terms of accuracy, sensitivity, specificity, precision, F1-score, and AUC. The proposed method produced 98.32% accuracy on the test set. The average sensitivity, specificity, precision, F1-score, and AUC were 96.61%, 98.56%, 98.68%, 97.65%, and 97.88%, respectively. Table 7 compares the proposed IMPA-ResNet50 model with the MPA-ResNet50 method based on manual search on the CBIS-DDSM dataset. According to the results presented in Table 7, the proposed IMPA-ResNet50 model outperforms ResNet50 model without the hyperparameters optimization. Where, the accuracy of the ResNet50 architecture is 90.11%, the sensitivity is 89.80%, specificity is 90.33%, the precision, F1 score, and AUC of this architecture are 89.01%, 90.00%, 91.88%, respectively. The improvement of the proposed IMPA-ResNet50 model relative to the ResNet50 architecture according to the accuracy is 8.21%, and the improvement according to the sensitivity, specificity, precision, F1 score, and AUC are 6.81%, 8.23%, 9.67%, 7.65%, 6.00%, respectively.

Furthermore, the proposed IMPA-ResNet50 model’s performance was compared with other published studies on breast cancer diagnosis using the CBIS-DDSM dataset in Table 8. These studies [35, 46, 48, 74–79] were chosen for comparison because they are recent models and were trained on a various data samples. The proposed IMPA-ResNet50 model was compared with other studies in terms of accuracy, sensitivity, specificity, precision, F1-score, and AUC, as shown in Table 8. The symbol − in the table means the comparison method does not have an equivalent metric. In [35], several CNN models were introduced to classify three datasets. The significant performance was on the DDSM dataset that contains 5316 images and the ResNet50 model. The ResNet50 model achieves 97.27% accuracy. In [76], multi-deep CNN models were evaluated on two datasets: CBIS-DDSM and MIAS. They used 5272 images from the first dataset and achieved accuracy, Sensitivity, and specificity of 87.2%, 86.04%, and 89.40%, respectively. The study in [48] proposed a fine-tuned technique to enhance the performance of the ResNet50 model. This method was evaluated on 2620 images from the CBIS-DDSM dataset and achieved accuracy, specificity, Sensitivity, AUC of 93.15%, 92.17%, 93.83%, 0.95, respectively. While in [46], the authors hybridized the SVM and the CNN on CBIS-DDSM dataset to enhance the performance. The obtained accuracy was 87.2% as shown in Table 8. In addition, in [74], 600 images from the DDSM dataset have been classified and achieved 96.7% accuracy. In [75], a YOLO-CNN method was proposed for classifying 2400 images from DDSM dataset as seen in row 5 of Table 8. Several CNN models used in [77] to classify three breast cancer datasets. A DCNN model proposed in [78] uses a large number of images; 11562 images. The gained accuracy is 92.80%. According to the table, the proposed IMPA-ResNet50 model’s classification performance is superior to other approaches. It outperforms all comparison methods in terms of all evaluation matrices.

**Table 6:** Results of the proposed IMPA-ResNet50 on the CBIS-DDSM dataset

Metrics	IMPA-ResNet50 (%)
Accuracy	98.32
Sensitivity	96.61
Specificity	98.56
Precision	98.68
F-score	97.65
AUC	97.88

**Table 7:** Comparison between the proposed IMPA-ResNet50 model and ResNet50 model on the CBIS-DDSM dataset

Metrics	ResNet50 (%)	IMPA-ResNet50 (%)	Improvement (%)
Accuracy	90.11	98.32	8.21
Sensitivity	89.80	96.61	6.81
Specificity	90.33	98.56	8.23
Precision	89.01	98.68	9.67
F1-score	90.00	97.65	7.65
AUC	91.88	97.88	6.00

**Table 8:** Comparison between the proposed IMPA-ResNet50 model and other related studies on the CBIS-DDSM dataset

References	No. of images	Dataset	Model	Accuracy (%)	Sensitivity (%)	Specificity	Precision (%)	F1-score (%)	AUC
[35]	5316	DDSM	ResNet50	97.35	–	–	–	–	0.97
[35]	5316	DDSM	VGG16	97.12	–	–	–	–	0.98
[74]	600	DDSM	CNN	96.7	–	–	–	–	–
[75]	2400	DDSM	CNN-YOLO	97.0	93.20	94.00	–	–	96.45%
[48]	2620	CBIS-DDSM	Fine-tuned ResNet50	93.15	93.83	92.17%	–	–	95.0%
[76]	5272	CBIS-DDSM	ResNet50	87.2	86.04	89.40%	–	–	95.00%
[46]	5272	CBIS-DDSM	Fine-tuned AlexNet	87.20	86.2	87.7%	88.0	87.1	94.00%
[77]	3568	CBIS-DDSM	ResNet50	96.6	92.95	88.60%	–	–	93.4%
[78]	11,562	DDSM	DCNN	92.80	–	–	–	–	–
[79]	2781	CBIS-DDSM	AdaBoost	90.91	82.96	98.38%	86.00	–	98.32%
Proposed	5283	CBIS-DDSM	IMPA-ResNet50	98.32	95.61	98.56%	98.68	97.65	97.88%

### Analysis of IMPA-ResNet50 model for MIAS dataset

This subsection presents the results of the proposed method IMPA-ResNet50 using hyperparameters calculated by the IMPA algorithm according to the MIAS dataset. Furthermore, it presents the comparison between other related studies. In addition, to demonstrate the effectiveness of the IMPA in determining the best values for the hyperparameters of the ResNet50 model that can achieve the most significant accuracy, we compare it with the ResNet50 model based on setting the hyperparameters of the ResNet50 model manually, i.e., randomly chosen without optimization.

Table 9 presents the results of IMPA-ResNet50 for the MIAS dataset evaluated in terms of accuracy, sensitivity, specificity, precision, F1-score, and AUC. The proposed method achieved 98.88% accuracy. The average sensitivity, specificity, precision, F1-score, and AUC were 97.61%, 98.40%, 98.30%, 97.10%, and 99.24%, respectively. Table 10 compares IMPA-ResNet50 model with the MPA-ResNet50 method based on manual search for the MIAS dataset. According to the results in Table 10, the proposed ResNet50 model outperform the ResNet50 architecture that selected without the hyperparameters optimization. Where, the accuracy of the ResNet50 architecture is 87.50%, the sensitivity is 88.10%, specificity is 86.12%, the precision, F1 score, and AUC of this architecture are 87.32%, 87.88%, 89.01%, respectively. The improvement of the proposed IMPA-ResNet50 model relative to the ResNet50 architecture according to the accuracy is 11.38%, and the improvement according to the sensitivity, specificity, precision, F1 score, and AUC are 9.51%, 12.28%, 10.98%, 9.22%, 10.23%, respectively.

In addition, the proposed IMPA-ResNet50 model’s performance was compared with other published studies on breast cancer diagnosis using the MIAS dataset. The comparison studies, [35, 36, 43, 76, 80, 81], were chosen because they are based on CNN architectures and have used the same dataset. The methods were compared in terms of accuracy, sensitivity, specificity, precision, F1-score, and AUC, as shown in Table 11. The symbol - in the table means the comparison method does not have the equivalent metric. In [35], several CNN models were introduced to classify three datasets. The obtained results on the MIAS dataset are 98.23% accuracy and 0.99 AUC. In [36], the graph convolutional network is used with CNN to classify 322 images; the results of that method are reported in row 4 on Table 11. In [43], an improved CNN model was introduced to classify the MIAS breast cancer dataset that achieves 89.47%, 90.71%, and 90.50% for sensitivity, specificity, and accuracy, respectively. In [80], a DenseNet201 model evaluated on the MIAS dataset and obtain 92.73% accuracy. Based on the table, the proposed IMPA-ResNet50 model is superior to other approaches. It outperforms all comparison methods in terms of all evaluation matrices.

**Table 9:** Results of the proposed IMPA-ResNet50 on the MIAS dataset

Metrics	IMPA-ResNet50 (%)
Accuracy	98.88
Sensitivity	97.61
Specificity	98.40
Precision	98.30
F-score	97.10
AUC	99.24

**Table 10:** Comparison between the proposed IMPA-ResNet50 model and the ResNet50 model on the MIAS dataset

Metrics	ResNet50 (%)	IMPA-ResNet50 (%)	Improvement (%)
Accuracy	87.50	98.88	11.38
Sensitivity	88.10	97.61	9.51
Specificity	86.12	98.40	12.28
Precision	87.32	98.30	10.98
F1-score	87.88	97.10	9.22
AUC	89.01	99.24	10.23

**Table 11:** Comparison between the proposed IMPA-ResNet50 model and other related studies on the MIAS dataset

References	No. of images	Dataset	Model	Accuracy (%)	Sensitivity (%)	Specificity (%)	Precision (%)	F1-score (%)	AUC (%)
[35]	322	MIAS	ResNet50	98.23	–	–	–	–	99.0
[76]	1288	MIAS	Fine-tuned DCNN	95.4	96.60	92.10	–	–	99.00
[36]	322	MIAS	CNN-GCN	96.10 ± 1.60	96.20 ± 2.90	96.00 ± 2.30	–	–	–
[80]	330	MIAS	DenseNet201	92.73	94.58	91.67	–	–	–
[81]	322	MIAS	CNN	82.68	82.73	82.71	–	–	–
[43]	322	MIAS	CNN	89.47	90.71	90.50	–	–	–
Proposed	1290	MIAS	IMPA-ResNet50	98.88	97.61	98.40	98.30	97.10	99.24

### Comparison with other optimization algorithms

This subsection presents the comparative results between the IMPA algorithm and other well-known meta-heuristic algorithms to demonstrate that the IMPA algorithm effectively determines the best values for the ResNet50 architecture’s hyperparameters to reach high accuracy. It was compared to four other meta-heuristic algorithms that have received much attention and highly cited meta-heuristics: the GSA algorithm [82], the HHO algorithm [83], the WOA algorithm [84], and the original MPA algorithm. These algorithms are also hybrid with the ResNet50 architecture produce models named GSA-ResNet50, HHO-ResNet50, WOA-ResNet50, and MPA-ResNet50, respectively. For a fair comparison between the IMPA and the other compared algorithms, all the compared algorithms have the same parameters as the IMPA, as stated in Table 5. Table 12 presents the comparison between the proposed IMPA-ResNet50 model with the MPA-ResNet50, GSA-ResNet50, HHO-ResNet50, and WOA-ResNet50 models. According to the comparative results, the IMPA is more suitable for combining with the ResNet50 architecture to classify the mammography breast cancer datasets. It was able to select the optimal hyperparameter values for the ResNet50, resulting in a greater accuracy ratio for this architecture.

In summary, the following observations from the reported experiments are worth mentioning

- *In terms of accuracy* It is observed that the proposed IMPA-ResNet50 model outperforms the MPA-ResNet50 model, which means the improved version of the MPA achieves a significant performance when used to optimize the ResNet50 hyperparameters compared to the original MPA algorithm. The MPA-ResNet50 model achieves an accuracy of 95.59% on the CBIS-DDSM dataset and 94.95% on the MIAS dataset. Also, the IMPA-ResNet50 model outperforms all other compared models. In the case of using the GSA algorithm to select the hyperparameters of the ResNet50, it achieves an accuracy of 95.48% on the CBIS-DDSM dataset and 94.38% on the MIAS dataset. In comparison, the HHO-ResNet50 model achieves an accuracy of 94.55% on the CBIS-DDSM dataset and 94.30% on the MIAS dataset. While the WOA-ResNet50 model achieves an accuracy of 94.13% on the CBIS-DDSM dataset and 93.38% on the MIAS dataset.
- *In terms of sensitivity, specificity, precision, and F-score* As listed in Table 12, the performance of the proposed IMPA-ResNet50 model outperforms all other compared models, which means the IMPA algorithm is very appropriate in conjunction with the ResNet50 architecture.

**Table 12:** Comparison between the proposed IMPA-ResNet50 model with the MPA-ResNet50, GSA-ResNet50, HHO-ResNet50, and WOA-ResNet50 models

Dataset	Classification Model	Accuracy (%)	Sensitivity (%)	Specificity (%)	Precision (%)	F-score (%)
CBIS-DDSM	IMPA-ResNet50	98.32	96.61	98.56	98.68	97.65
	MPA-ResNet50	95.95	93.03	95.28	94.22	93.85
	GSA-ResNet50	95.48	94.16	95.00	95.00	94.00
	HHO-ResNet50	94.55	93.12	94.84	94.12	94.50
	WOA-ResNet50	94.13	93.10	94.00	94.00	94.00
MIAS	IMPA-ResNet50	98.88	97.61	98.40	98.30	97.10
	MPA-ResNet50	94.95	94.03	94.28	94.22	94.85
	GSA-ResNet50	94.38	94.00	93.38	94.16	94.00
	HHO-ResNet50	94.30	93.50	94.18	93.69	94.00
	WOA-ResNet50	93.38	93.00	93.00	93.00	93.00

### Limitations of the proposed model

This paper proposes an efficient breast cancer classification model that depends on the hybridization of pretrained CNN architecture and an improved meta-heuristic optimization algorithm. Although the proposed IMPA-ResNet50 model achieves high classification performance in breast cancer detection from mammography images, future studies need to address some limitations. The limitation of the IMPA algorithm and the limitation of the proposed IMPA-ResNet50 model are illustrated as follows:

- Because the IMPA algorithm is merged with the OBL strategy, it is comparatively computationally expensive than the original MPA algorithm.
- The running time may increase when adding the OBL, but the increase in the time here is for getting more performance compared to the original algorithm.
- The IMPA-ResNet50 was only implemented to classify mammography images. These results are limited to a specific dataset, MIAS dataset, and CBIS-DDSM dataset and may not be generalized to the other dataset.
- The performance of the IMPA-ResNet50 is closely related to medical imaging applications.
- The IMPA algorithm success in determining the values of the hyperparameters of the ResNet50 architecture only, and it may not be generalized to other pretrained CNN architecture.

## Conclusion and future work

Deep Learning is one of the essential techniques in medical imaging classification. Convolution Neural Networks are examples of common DL techniques used for biomedical image classification that involves layer-wise automatic feature extraction. The preparation of CNNs for classification objectives depends on the knowledge of hyperparameter tuning. Hyperparameters for each layer are different. Because these hyperparameters determine a CNN model’s performance, they need to be fine-tuned to achieve great results. It is not suitable to optimize hyperparameters manually because manually selecting them is quite a complex and time-consuming task. Meta-heuristic algorithms have significantly influenced hyperparameter optimization in several fields. This paper proposes a novel classification model for breast cancer based on a hybridized pretrained CNN architecture (ResNet50) and an improved meta-heuristic optimization algorithm. The marine predators algorithm (MPA) is the used optimization algorithm, and we improve it using the OBL strategy to improve exploitation and avoid getting stuck in the local optimal. The Improved Marine Predators Algorithm (IMPA) is used to find the best values for the hyperparameters of the ResNet50 architecture, resulting in a model called IMPA-ResNet50. To the best of our knowledge, this is the first paper using the IMPA as an optimization algorithm to optimize the hyperparameters for the ResNet50 architecture for breast cancer classification. The proposed IMPA-ResNet50 model includes four phases: (1) the data preprocessing and augmentation phase, (2) hyperparameter optimization phase, (3) learning phase, and (4) performance evaluation phase. The proposed model is compared with other state-of-the-art methods and other CNN approaches. The comparison results showed the effectiveness of the proposed model in diagnosing breast cancer. The evaluation is performed on two mammography datasets: the curated breast imaging subset of DDSM (CBIS-DDSM) and the mammographic image analysis society (MIAS). To demonstrate the effectiveness of the IMPA in determining the best values for the hyperparameters of the ResNet50 model that can achieve the most significant accuracy, we compare it with the MPA-ResNet50 model, also compare it with the pretrained CNN ResNet50 model based on the manual search. The first experiment applied to the CBIS-DDMS consisted of 5283 images for two classes, normal and abnormal. The obtained accuracy was 98.32% in the testing phase according to the IMPA-ResNet50 model and 95.95% accuracy according to the MPA-ResNet50 model, which means the improved version for the MPA (i.e., IMPA) significantly improves performance when used to optimize the ResNet50 hyperparameters compared to the original MPA. The second experiment was performed using another dataset (MIAS) consisting of 1290 images after applying the data augmentation techniques. The obtained accuracy was 98.88% according to IMPA-ResNet50 95.95% accuracy according to MPA-ResNet50. Also, compared to four meta-heuristic algorithms: GSA, HHO, WOA, and the original MPA algorithm, the results of the comparison showed the effectiveness of the proposed algorithm. The proposed method’s performance has been assessed using five measures: accuracy, sensitivity, specificity, precision, and F-score. The results showed that the proposed model gets better results than the other competing algorithms, which means using the IMPA as a metaheuristic optimization algorithm to determine the ResNet50 architecture’s hyperparameters boosts the classification models to achieve the best performance for a breast cancer diagnostic.

In future work, multiple datasets with more images will be used and evaluated in the proposed model. Also, various pre-trained models such as DensNet201, DensNet121, and Inception will be used to classify breast cancer in conjunction with the proposed IMPA algorithm. In addition, various metaheuristic algorithms will be used for hyperparameter tuning. Also, we will test the proposed method’s performance in solving different medical image classification problems and different diagnosis applications, applying the IMPA algorithm to solving other medical and engineering issues, and using it as a feature selection method. Furthermore, various feature extraction methods will be used with CNN architecture to improve the classification accuracy further. Transfer learning with other models can enhance the performance.
